# Effect of magnesium sulfate perioperative infusion on postoperative catheter-related bladder discomfort in male patients undergoing laparoscopic radical resection of gastrointestinal cancer: a prospective, randomized and controlled study

**DOI:** 10.1186/s12871-023-02346-z

**Published:** 2023-12-02

**Authors:** Wencai Jiang, Xu Zeng, Xinyu Zhou, Ou Liao, Feng Ju, Zhifu Zhao, Xianjie Zhang

**Affiliations:** 1 Department of Anesthesiology, Deyang People’s Hospital, Deyang, 618000 China; 2 Clinical Medicine Department, Chengdu Medical College, Chengdu, 610500 China

**Keywords:** Magnesium sulfate, Catheter-related bladder discomfort, Gastrointestinal cancer

## Abstract

**Background:**

Laparoscopic radical resection of gastrointestinal cancer is associated with a high incidence of postoperative catheter-related bladder discomfort (CRBD). Studies on the benefits of magnesium sulfate intravenous infusion during the perioperative period post-laparoscopic surgery are yet lacking.

**Methods:**

A total of 88 gastrointestinal cancer male patients scheduled for laparoscopic radical resection were randomly divided into two groups: normal saline (control) and magnesium. In the magnesium group, a 40 mg/kg loading dose of intravenous magnesium sulfate was administered for 10 min just after the induction of anesthesia, followed by continuous intravenous infusion of 15 mg/kg/h magnesium sulfate until the end of the surgery; the control group was administered the same dose of normal saline. Subsequently, 2 μg/kg sufentanil was continuously infused intravenously by a postoperative patient-controlled intravenous analgesia (PCIA) device. The primary outcome was the incidence of CRBD at 0 h after the surgery. The secondary outcomes included incidence of CRBD at 1, 2, and 6 h postsurgery, the severity of CRBD at 0, 1, 2, and 6 h postsurgery. Remifentanil requirement during surgery, sufentanil requirement within 24 h postsurgery, the postoperative numerical rating scale (NRS) score at 48 h after the surgery, magnesium-related side effects and rescue medication (morphine) requirement were also assessed.

**Results:**

The incidence of CRBD at 0, 1, 2, and 6 h postoperatively was lower in the magnesium group than the control group (0 h: *P* = 0.01; 1 h: *P* = 0.003; 2 h: *P* = 0.001; 6 h: *P* = 0.006). The incidence of moderate to severe CRBD was higher in the control group at postoperative 0 and 1 h (0 h: *P* = 0.002; 1 h: *P* = 0.028), remifentanil requirement during surgery were significantly lower in the magnesium group than the control group. Sufentanil requirements during the 24 h postoperative period were significantly lower in the magnesium group than the control group. The NRS score was reduced in the magnesium group compared to the control group in the early postoperative period. Magnesium-related side effects and rescue medication (morphine) did not differ significantly between the two groups.

**Conclusions:**

Intravenous magnesium sulfate administration reduces the incidence and severity of CRBD and remifentanil requirement in male patients undergoing radical resection of gastrointestinal cancer. Also, no significant side effects were observed.

**Trial registration:**

Chictr.org.cn ChiCTR2100053073. The study was registered on 10/11/2021.

## Background

Gastrointestinal cancers have a high incidence, economic burden, and mortality in China, accounting for 45% of all cancer deaths in 2020 [1]. With the development of enhanced recovery after surgery (ERAS), laparoscopic resection has become the preferred method of gastrointestinal cancer treatment due to its characteristics of enhanced recovery and fewer postoperative complications [2, 3]. In order to facilitate intraoperative management and postoperative recovery, patients with gastrointestinal cancer radical resection require preoperative indwelling urinary catheters, which might cause patients to develop catheter-related bladder discomfort (CRBD) after awakening from general anesthesia.

CRBD refers to symptoms such as frequent urination, urgent urination, and discomfort in the suprapubic area due to catheter stimulation [4–6]. The severity of CRBD was recorded as follows: None, patients complained of no bladder discomfort when asked; Mild, patients complained of slight urethral discomfort only when they were asked; Moderate, patients actively expressed urethral discomfort when they were not asked but were able to comply with movements without abnormal physical behavior; Severe, patients were not asked, and they actively expressed urinary urge, urethral burning, or foreign body sensation accompanied by behavioral reactions such as emotional irritability and limb movement [6].CRBD often occurs in the early postoperative period, especially in the post-anesthesia care unit (PACU) [6]. Some drugs, such as gabapentin, ketamine, and tramadol, have shown varying degrees of success in the prevention of CRBD, however, the incidence of CRBD still reaches 47–90% [7–10]. It is a painful complication, but it is often neglected in the clinic. It can exacerbate postoperative pain and restlessness, leading to serious adverse events. Therefore, clinical staff must focus on the early prevention and active treatment of CRBD [11–13]. Presently, a regimen for the management of CRBD is not yet established.

Magnesium participates in the active transport of calcium, inhibiting the release of neurotransmitters in the body and relaxing the smooth muscles [14]. It was reported that magnesium sulfate reduces the severity of CRBD in patients who have undergone transurethral resection of bladder tumor [15], but its applicability needs to be investigated further. Male, catheter diameter, bladder-related surgery, and lack of additional analgesics are risk factors for the development of CRBD [16, 17]. To explore ways to reduce the incidence of CRBD in male patients and distinguish CRBD from spasms or pain associated with genitourinary surgeries, we selected male patients undergoing laparoscopic surgeries for the present study.

We hypothesized that perioperative administration of magnesium sulfate might decrease the incidence and severity of CRBD. The present study aimed to assess the efficacy of magnesium sulfate perioperative infusion to reduce the incidence and severity of CRBD in male patients undergoing laparoscopic radical resection of gastrointestinal cancer.

## Methods

### Study design and population

This study was approved by the Ethics Committee of the Deyang People’s Hospital on January 26, 2022 (No. 2022–04-009-K01). This single-center, double-blinded, prospective, randomized study was registered with China Clinical Trial Registration on 10/11/2021 (ChiCTR2100053073). All male patients were enrolled between March and September 2022, and each patient provided written informed consent before entering the trial.

Patients scheduled for elective laparoscopic radical gastrointestinal cancer surgery during March ~ September 2022 were screened through the surgery scheduling system of Deyang People’s Hospital. Eligible patients were 18–70 years old, American Society of Anesthesiologists (ASA) physical status I to III, scheduled for elective laparoscopic radical gastrointestinal cancer surgery (including Gastric surgery, Right Hemicolectomy, Left Hemicolectomy, Sigmoidectomy, and Rectal surgery) using 14 or 16 French (Fr) Foley catheters inserted. Then, we were extubated in the operating room. Patients were excluded if they had hypermagnesemia risk (chronic kidney disease, primary hyperparathyroidism, hyperaldosteronism, and abnormal liver function), contraindications (myocardial damage, atrioventricular block, and allergy to magnesium sulfate) and taking medications known to interact with magnesium sulfate (lithium intake). Elimination criteria were changing to operation method, stay in ICU after the operation, and the first indwelling catheter was failure.

### Randomization and blinding

According to a computer-generated (Microsoft Excel) random number table, participants were randomly assigned at 1:1 ratio to the two groups. The patients were unaware of the group allocation as this assignment was concealed in an opaque and sequentially numbered envelope. An investigator not involved in the trial opened the envelope to the attending anesthetists responsible for preparing the magnesium sulfate or normal saline. All data were acquired by the second investigator blinded to the group assignment. The statisticians and staff were also blinded to the treatment assignments in the ward and PACU.

### Interventions and anesthesia

Standardized monitoring included temperature, 3-lead electrocardiography, SpO2, blood pressure, end-tidal carbon dioxide (ETCO_2_), train-of-four (TOF) system, and bispectral index (BIS). General anesthesia was induced using midazolam (0.04 mg/kg), sufentanil (0.3–0.5 μg/kg), propofol (1–1.5 mg/kg), and rocuronium (0.6–1 mg/kg). Once the patient was unconscious, urinary catheterization was inserted using 14 or 16 French Foley catheters, and the balloon was inflated with 10 mL of distilled water. Anesthesia was maintained with a continuous target-controlled infusion of propofol (Marsh pharmacokinetic parameters: effect chamber target concentration Ce = 2.0 μg/mL) and additional sufentanil as needed. The degree of muscle blockade monitored by TOF-Watch SX, confirmed TOF count of at least 2. Rocuronium was intermittently administered to maintain muscle relaxation according to the TOF count. The depth of anesthesia was adjusted to the appropriate level according to the surgical requirements, and the BIS was maintained at 40–60. Intraoperative vasoactive drugs were used as necessary to maintain heart rate and blood pressure fluctuations within ± 20% of basal levels, followed by 30 mg of ketorolac and 5 mg of toltestrone 30 min before the end of the surgery. The PCIA device was attached to each patient at the end of surgery, administering 100 μg sufentanil, 10 mg toltestrone, and 90 mg ketorolac in 100 mL of normal saline to deliver 2 mL/h as the basal infusion and 2 mL per demand with a 15-min lockout period. After confirming that the patient was fully conscious (BIS of at least 90) and had recovered from neuromuscular blockade (TOF ratio of at least 90%), the endotracheal tube was removed, and the patient was transferred to the PACU.

In the magnesium group, a 40 mg/kg loading dose of intravenous magnesium sulfate was administered for 10 min immediately after the induction of anesthesia, followed by continuous intravenous infusion of 15 mg/kg/h magnesium sulfate until the end of the surgery, and the control group was administered an equivalent volume of normal saline.

In the PACU and general ward, 2 μg sufentanil was administered if the patient complained of moderate to severe CRBD or postoperative pain (NRS ≥ 4). Then, the patient was reassessed 5 min after drug administration, and 2 mg of morphine was administered as a rescue agent if the patient requested additional analgesia.

### Outcomes

The primary outcome was the incidence of CRBD at 0 h after the surgery (on admission to the PACU). The secondary outcomes included incidence of CRBD at 1, 2, and 6 h postsurgery, the severity of CRBD at 0, 1, 2, and 6 h postsurgery. Other outcomes included remifentanil requirement during surgery, sufentanil requirement within 24 h postsurgery, the NRS score at 48 h after the surgery, magnesium-related side effects and rescue medication (morphine) requirement.

Magnesium-related adverse effects, such as hypotension, bradycardia, respiratory depression, agitation, shivering, dry mouth, nausea and vomiting, were assessed intraoperatively and at 6 h postoperatively. Hypotension and bradycardia were defined as decrease of mean arterial pressure and heart rate by 20% of baseline value. Depression, agitation, shivering, dry mouth, nausea and vomiting were reported by the staff or patient in a yes or no question.

### Sample size and statistical analysis

Sample size calculation was performed using the PASS 15.0 (NCSS LLC., Kaysville, U.T., USA). The current results showed that CRBD occurred in 70% of male patients undergoing laparoscopic radical resection of gastrointestinal cancer (based on our unpublished pilot data). Assuming a rounded 40% rate of CRBD in the magnesium group, we calculated a total sample size of 104 male patients (52 in each group) with a two-sided alpha level of 0.05, a power of 0.8, and a 20% dropout.

The data are expressed as mean ± standard deviation, median (interquartile range, IQR), number (proportion), relative risk (RR), and 95% confidence interval (95% CI). Continuous data were checked for normality using the Shapiro–Wilk test. The continuous normally distributed variables were compared using Student’s t-test; whereas the Mann–Whitney and Wilcoxon tests were used for non-normally distributed variables. Two or more proportions were compared using the chi-square test or Fisher’s exact test as appropriate. Repeated measures ANOVA was used for analyzing the differences between variables over a period. Statistical analysis was carried out using SPSS v26.0 (IBM Corp., Armonk, N.Y., USA).

## Results

**Fig. 1:**
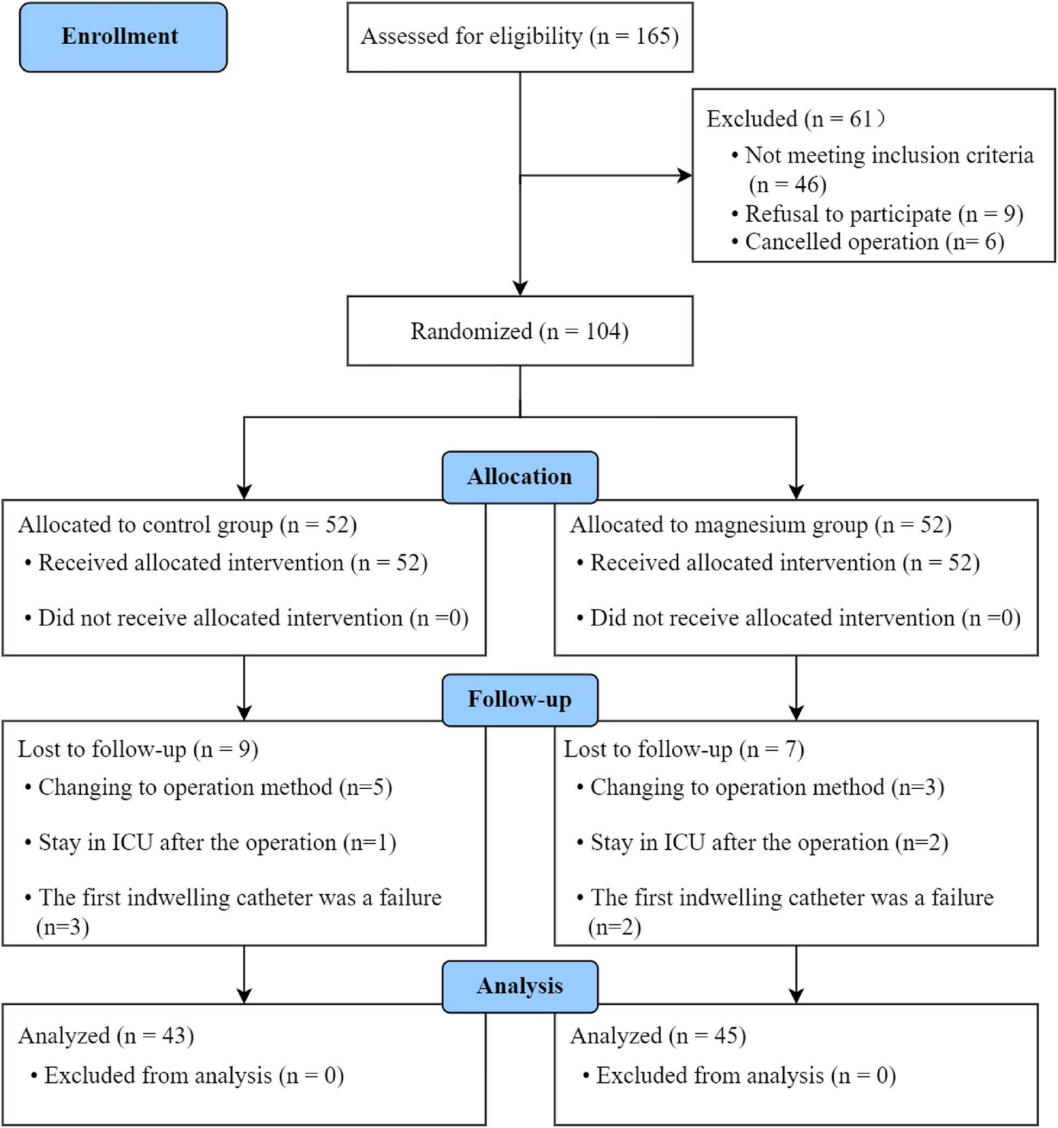
Schematic of the progress through the phases of the trial. ICU, intensive care unit

A total of 165 male patients were assessed for eligibility before surgery. Among them, 61 male patients were excluded, and 104 were randomized (Fig. 1). After randomization, 9 male patients in the control group were lost to follow-up because their operation was changed, they were transferred to the intensive care unit (ICU), or the first indwelling was a failure, while 7 male patients in the magnesium group were lost to follow-up due to the same reasons. Baseline preoperative and intraoperative characteristics (not including intraoperative remifentanil requirement) did not differ between the two groups (Tables 1 and 2), remifentanil requirement during surgery were lower in the magnesium group than the control group (0.9 ± 0.3 *vs*. 0.7 ± 0.2, *P* < 0.001).

**Table 1:** Preoperative baseline characteristics

	Control group (*n* = 43)	Magnesium group (*n *= 45)	*P*-value
Age (years)	60.7 ± 8.0	61.0 ± 7.1	0.850
Height (cm)	164.7 ± 5.2	165.1 ± 7.6	0.792
Weight (kg)	62.5 ± 7.8	63.5 ± 8.8	0.574
Body mass index (kg/m^2^)	23.0 ± 2.7	23.3 ± 2.4	0.689
ASA classification (n)			0.853
I/II/III	0/19/24	0/19/26	
Type of surgery			0.647
Gastric surgery	10 (23.3)	9 (20.0)	
Right Hemicolectomy	4 (9.3)	6 (13.3)	
Left Hemicolectomy	4 (9.3)	2 (4.4)	
Sigmoidectomy	10 (23.3)	7 (15.6)	
Rectal surgery	15 (34.9)	21 (46.7)	
Urinary catheter size (Fr) (n)			0.311
14/16	32/11	29/16	

Data are expressed as mean ± SD or number or numbers (%)

**Table 2:** Intraoperative characteristics

	Control group (*n* = 43)	Magnesium group (*n* = 45)	*P*-value
Duration of surgery (min)	201.7 ± 48.4	210.8 ± 47.2	0.372
Time to extubation (min)	15.6 ± 5.0	14.7 ± 4.0	0.368
Intra-operative rocuronium requirement (mg)	95.6 ± 23.9	89.6 ± 21.4	0.213
Intra-operative sufentanil requirement (μg)	35.6 ± 5.5	35.9 ± 5.2	0.820
Intra-operative remifentanil requirement (mg)	0.9 ± 0.3	0.7 ± 0.2	< 0.001
Intra-operative propofol requirement (mg)	1286.5 ± 330.3	1339.8 ± 298.2	0.429
Urine output (mL)	200 (150–300)	200 (150–400)	0.614
Blood loss (mL)	50 (30–100)	50 (25–100)	0.601
Total fluids (mL)	1300 (1100–1600)	1200 (1200–1400)	0.444

Data are expressed as mean ± SD or M (IQR)

The incidence of CRBD at 0, 1, 2, and 6 h postoperatively was lower in the magnesium group than the control group (0 h: 18 [40%] *vs*. 29 [67.4%], *P* = 0.01; RR = 0.54, 95% CI: 0.33–0.88; 1 h: 17 [37.8%] *vs*. 30 [69.8%], *P* = 0.003; RR = 0.48, 95% CI: 0.29–0.81; 2 h: 8 [17.8%] *vs*. 22 [51.2%], *P* = 0.001; RR = 0.59, 95% CI: 0.42–0.83; 6 h: 4 [8.9%] *vs*. 14 [32.6%], *P* = 0.006; RR = 0.74, 95% CI: 0.59–0.92). The incidence of moderate to severe CRBD was higher in the control group at postoperative 0 and 1 h (0 h: 62.1% *vs*. 16.7%, *P* = 0.002; 1 h: 56.7% *vs*. 23.5%, *P* = 0.028). The incidence of moderate-to-severe CRBD did not differ significantly between the two groups at 2 and 6 h (2 h: 31.8% *vs*. 12.5%, *P* = 0.391; 6 h: 0% *vs*. 0%, *P* = 1.000) (Table 3). A significant difference was observed in the NRS scores in the comparison between the control and magnesium groups at 0, 1, 2, and 6 h (*P* < 0.05).

**Table 3:** Incidence and severity of catheter-related bladder discomfort

Postoperative	0 h		1 h		2 h		6 h	
group	Control (*n* = 43)	Mag (*n* = 45)	Control (*n* = 43)	Mag (*n* = 45)	Control (*n* = 43)	Mag (*n* = 45)	Control (*n* = 43)	Mag (*n* = 45)
Incidence (%)	29 (67.4)	18 (40.0)	30 (69.8)	17 (37.8)	22 (51.2)	8 (17.8)	14 (32.6)	4 (8.9)
P-value	0.010		0.003		0.001		0.006	
Relative risk (95% CI)	0.54 (0.33–0.88)		0.48 (0.29–0.81)		0.59 (0.42–0.83)		0.74 (0.59–0.92)	
Severity								
Mild	11 (37.9)	15 (83.3)	13 (43.3)	13 (76.5)	15 (68.2)	7 (87.5)	14 (100)	4 (100)
Moderate to severe	18 (62.1)	3 (16.7)	17 (56.7)	4 (23.5)	7 (31.8)	1 (12.5)	0 (0)	0 (0)
P-value	0.002		0.028		0.391		1.000	

Data are expressed as numbers (%). Mag, Magnesium

Compared to the control group, no significant differences were observed in the NRS score within 12, 24, and 48 h after surgery (*P* > 0.05). Sufentanil requirements during the 24-h postoperative period were significantly lower in the magnesium group than in the control group [50 μg (IQR, 50–52 μg) *vs*. 54 μg (IQR, 52–56 μg), *P* < 0.001]. No significant differences were detected in postoperative morphine requirements and other side effects between the control and magnesium groups (20.9% *vs*. 8.9%, *P* = 0.134) within 24 h postoperatively (Tables 4 and 5).

**Table 4:** NRS score within 48 h and analgesia requirement within 24 h after surgery

Postoperative group	Control group (*n* = 43)	Magnesium group (*n* = 45)	*P*-value
0 h	2 (1–2)	1 (0–2)	0.013
1 h	3 (2–3)	2 (1–3)	0.032
2 h	2 (2–3)	2 (1–2)	0.008
6 h	2 (2–2)	2 (1–2)	0.007
12 h	2 (1–2)	1 (1–2)	0.079
24 h	1 (1–2)	1 (1–2)	0.374
48 h	1 (1–1)	1 (1–1)	0.584
Cumulative use of sufentanil at 24 h (μg)	54 (52–56)	50 (50–52)	< 0.001
Rescue analgesia	9 (20.9)	4 (8.9)	0.134

Data are expressed as M (IQR) or numbers (%)

**Table 5:** Incidence of side effects

	Control group (*n* = 43)	Magnesium group (*n* = 45)	*P*-value
Intraoperative			
Bradycardia	14 (32.6)	8 (17.8)	0.109
Hypotension	7 (16.3)	8 (17.8)	0.852
Postoperative			
Respiratory depression	1 (2.3)	1 (2.2)	1.000
Agitation	4 (9.3)	2 (4.4)	0.429
Shivering	0 (0)	1 (2.2)	1.000
Dry mouth	1 (2.3)	1 (2.2)	1.000
Nausea/vomiting	3 (7.0)	2 (4.4)	0.673

Data are expressed as numbers (%)

## Discussion

In this randomized controlled trial, magnesium sulfate administration significantly decreased the incidence and severity of CRBD and remifentanil requirement among male patients with laparoscopic radical resection of gastrointestinal cancer. In addition, the early postoperative NRS score of the control group was higher than that of the magnesium group. No significant differences were observed in magnesium-related adverse effects.

The discomfort and abnormal behavioral responses caused by CRBD often lead to agitation during the awakening period, which in turn leads to increased postoperative complications, such as dehiscence of the surgical incision, bleeding, circulatory instability, cardiac arrhythmias, prolonged hospital stay, and increased mortality during hospitalization [18–21]. CRBD have a more incidence in male than females [17], herein, in male patients undergoing laparoscopic radical resection of gastrointestinal cancer with a 14 or 16 Fr Foley catheter, the incidence of CRBD was 69.8%. Therefore, reducing the incidence and severity of postoperative CRBD is an integral part of perioperative anesthetic management.

Two different mechanisms may exist for CRBD. The first mechanism is that the catheter causes activation of urethral M receptors, which in turn leads to the contraction of urethral smooth muscle, resulting in CRBD [4, 22]. Therefore, dexmedetomidine, tolterodine, solifenacin, butylscopolamine, and trospium have been studied for the prevention of CRBD with varying success rates [11, 23–26], and some analgesics such as tramadol are used based on this mechanism [12, 18]. Another mechanism is the release of prostaglandins, such that many anti-inflammatory drugs, including lidocaine and ketorolac, have been reported for the prevention of CRBD [4, 13]. Although such interventions are carried out, the incidence of CRBD has the highest proportion.

In the prsent study, magnesium sulfate administration effectively reduced the incidence and severity of CRBD after laparoscopic radical resection of gastrointestinal cancer. However, intravenous magnesium sulfate administration has been associated with several concerns, including the risk of neuromuscular and cardiovascular effects. The recommended doses in the treatment of eclampsia are loading doses ranging from 4–6 g, and the maintenance dose was 1–2 g/h, which maintains the plasma concentrations at < 3.5 mmol/L to avoid magnesium poisoning [27]. No side effects associated with magnesium sulfate were reported; the same doses used in previous studies also did not report any associated side effects [15, 28]. Therefore, we recommended that intravenous magnesium sulfate is safe in male patients undergoing laparoscopic radical gastrectomy for gastrointestinal cancer. The use of magnesium sulfate in this study was extremely low, and the effect of high doses of magnesium sulfate on postoperative CRBD can be investigated further.

In this study, we enrolled male patients who underwent laparoscopic radical resection of gastrointestinal cancer using a 14 or 16 French Foley catheter. The incidence of CRBD at 0, 1, 2, and 6 h postoperatively was 67.4%, 69.8%, 51.2%, and 32.6% in the control group. Intraoperative magnesium sulfate decreased the incidence of CRBD to 40%, 37.8%, 17.8%, and 8.9%, respectively. This result was similar to that reported previously [8, 29]. The incidence of moderate to severe CRBD was high in the control group at postoperative 0 and 1 h (62.1% *vs*. 16.7%, *P* = 0.002; 56.7% *vs*. 23.5%, *P* = 0.028, respectively). This finding showed that magnesium sulfate is effective for the prevention of CRBD in male patients at a high risk of bladder discomfort.

We observed that intravenous magnesium sulfate administration reduces sufentanil requirement and decreases the NRS score in male patients undergoing radical resection of gastrointestinal cancer. Although there is a statistically significant difference between the two interventions, it is not major clinical relevance. As our pain management is well-executed, intravenous magnesium sulfate did not further improvement in pain management. However, the difference in the remifentanil requirement (albeit small 0.2 mg) was clinically significant. Magnesium is the antagonist of the N-methyl-D-aspartate receptor [14, 30, 31], some studies have shown that intraoperative intravenous magnesium sulfate does not reduce postoperative pain scores [15, 30], while others reported that it reduces postoperative pain in patients [28, 32]. This phenomenon might be attributed to various administration and surgical methods. Moreover, intravenous magnesium sulfate can be used as an adjuvant analgesic.

Nevertheless, the current study has several limitations. First, we did not measure plasma magnesium concentrations and hence, could not determine the therapeutic concentrations. Second, it is a major concern that magnesium sulfate enhances muscle relaxation, and muscle relaxation monitoring was carried out in all our studies. The requirement of rocuronium in the magnesium group was lower than that in the control group; however, the difference was not statistically significant. Importantly, a large trial is required to clarify whether magnesium sulfate reduces the rocuronium requirement. Third, we could not determine the optimal timing and dosage of the drug, which needs to be proved by additional studies. Previous studies have shown that intraoperative intravenous magnesium sulfate is safe [15, 28, 32]. Hence, we speculated that intraoperative intravenous magnesium sulfate could be used to prevent CRBD in male patients without contraindications.

## Conclusions

Intravenous magnesium sulfate administration reduces the incidence and severity of CRBD and remifentanil requirement in male patients undergoing radical resection of gastrointestinal cancer. Also, no significant side effects were observed.

## Data Availability

The datasets generated are publicly available at Chictr.org.cn (ChiCTR2100053073), and available from the corresponding author on reasonable request.

## References

[1] Cao W, Chen HD, Yu YW (2021). Changing profiles of cancer burden worldwide and in China: a secondary analysis of the global cancer statistics 2020[J]. Chin Med J (Engl).

[2] Zeng YK, Yang ZL, Peng JS (2012). Laparoscopy-assisted versus open distal gastrectomy for early gastric cancer: evidence from randomized and nonrandomized clinical trials[J]. Ann Surg.

[3] Lei QC, Wang XY, Zheng HZ (2015). Laparoscopic versus open colorectal resection within fast track programs: an update meta-analysis based on randomized controlled trials [J]. J Clin Med Res.

[4] Kim DH, Park JY, Yu J (2020). Intravenous lidocaine for the prevention of postoperative catheter-related bladder discomfort in male patients undergoing transurethral resection of bladder tumors: a randomized, double-blind, controlled trial [J]. Anesth Analg.

[5] In CB, Lee SJ, Sung TY (2021). Effects of Chlorpheniramine maleate on catheter-related bladder discomfort in patients undergoing Ureteroscopic stone removal: a randomized double-blind study [J]. Int J Med Sci.

[6] Agarwal A, Raza M, Singhal V (2005). The efficacy of Tolterodine for prevention of catheter-related bladder discomfort: a prospective, randomized, placebo-controlled, double-blind study [J]. Anesth Analg.

[7] Hu B, Li C, Pan M (2016). Strategies for the prevention of catheter-related bladder discomfort: A PRISMA-compliant systematic review and meta-analysis of randomized controlled trials[J]. Medicine (Baltimore).

[8] Li S, Li P, Wang R, et al. Different interventions for preventing postoperative catheter-related bladder discomfort: a systematic review and meta-analysis[J]. Eur J Clin Pharmacol. 2022;78(6):897–906. 10.1007/s00228-021-03251-5. 10.1007/s00228-021-03251-5 35218404

[9] Jang EB, Hong SH, Kim KS (2020). Catheter-related bladder discomfort: how can we manage it? [J]. Int Neurourol J.

[10] Wang YT, Xiao C, Liu H (2021). Preoperative oral gabapentin in the management of postoperative catheter-related bladder discomfort in adults: a systematic review and meta-analysis [J]. Front Surg.

[11] Chen H, Wang B, Li Q (2020). Intravesical dexmedetomidine instillation reduces postoperative catheter-related bladder discomfort in male patients under general anesthesia: a randomized controlled study [J]. BMC Anesthesiol.

[12] Li S, Song L, Ma Y (2018). Tramadol for the treatment of catheter-related bladder discomfort: a randomized controlled trial [J]. BMC Anesthesiol.

[13] Park JY, Hong JH, Yu J, et al. Effect of Ketorolac on the Prevention of Postoperative Catheter-Related Bladder Discomfort in Patients Undergoing Robot-Assisted Laparoscopic Radical Prostatectomy: A Randomized, Double-Blinded, Placebo-Controlled Study[J]. J Clin Med. 2019;8(6):759. 10.3390/jcm8060759. 10.3390/jcm8060759 PMC6616938 31146434

[14] Gröber U, Schmidt J, Kisters K (2015). Magnesium in prevention and therapy [J]. Nutrients.

[15] Park J-Y, Hong JH, Kim D-H (2020). Magnesium and bladder discomfort after transurethral resection of bladder tumor [J]. Anesthesiology.

[16] Bai Y, Wang X, Li X (2015). Management of catheter-related bladder discomfort in patients who underwent elective surgery [J]. J Endourol.

[17] Li SY, Song LP, Ma YS (2020). Predictors of catheter-related bladder discomfort after gynaecological surgery [J]. BMC Anesthesiol.

[18] Agarwal A, Yadav G, Gupta D (2008). Evaluation of intra-operative tramadol for prevention of catheter-related bladder discomfort: a prospective, randomized, double-blind study [J]. Br J Anaesth.

[19] Imai H, Seino Y, Baba H (2020). Efficacy of a novel urinary catheter for men with a local anesthetic injection port for catheter-related bladder discomfort: a randomized controlled study [J]. J Anesth.

[20] Zhang GF, Guo J, Qiu LL (2019). Effects of dezocine for the prevention of postoperative catheter-related bladder discomfort: a prospective randomized trial [J]. Drug Des Devel Ther.

[21] J Xiong, X Chen , C Weng  (2019). Intra-operative Oxycodone Reduced Postoperative Catheter-Related Bladder Discomfort Undergoing Transurethral Resection Prostate. A Prospective, Double Blind Randomized Study[J]. Urol J.

[22] Kim HC, Hong WP, Lim YJ (2016). The effect of sevoflurane versus desflurane on postoperative catheter-related bladder discomfort in patients undergoing transurethral excision of a bladder tumour: a randomized controlled trial [J]. Can J Anaesth.

[23] Tijani KH, Akanmu NO, Olatosi JO (2017). Role of tolterodine in the management of postoperative catheter-related bladder discomfort: findings in a Nigerian teaching hospital [J]. Niger J Clin Pract.

[24] Zhang Z, Cao Z, Xu C (2014). Solifenacin is able to improve the irritative symptoms after transurethral resection of bladder tumors[J]. Urology.

[25] Nam K, Seo JH, Ryu JH (2015). Randomized, clinical trial on the preventive effects of butylscopolamine on early postoperative catheter-related bladder discomfort[J]. Surgery.

[26] Srivastava VK, Agrawal S, Deshmukh SA (2020). Efficacy of trospium for prevention of catheter-related bladder discomfort: a prospective, randomized, placebo-controlled, double-blind study[J]. Korean J Anesthesiol.

[27] Sibai BM (2004). Magnesium sulfate prophylaxis in preeclampsia: lessons learned from recent trials[J]. Am J Obstet Gynecol.

[28] Dehkordy ME, Tavanaei R, Younesi E (2020). Effects of perioperative magnesium sulfate infusion on intraoperative blood loss and postoperative analgesia in patients undergoing posterior lumbar spinal fusion surgery: A randomized controlled trial [J]. Clin Neurol Neurosurg.

[29] Zhou Z, Cui Y, Zhang X (2021). The efficacy and safety of antimuscarinics for the prevention or treatment of catheter-related bladder discomfort: a systematic review and meta-analysis of randomized controlled trials [J]. Perioper Med (Lond).

[30] Adhikary SD, Thiruvenkatarajan V, Mcfadden A (2021). Analgesic efficacy of ketamine and magnesium after laparoscopic sleeve gastrectomy: a randomized, double-blind, placebo-controlled trial [J]. J Clin Anesth.

[31] Zhong HY, Zhang WP (2018). Effect of intravenous magnesium sulfate on bupivacaine spinal anesthesia in preeclamptic patients [J]. Biomed Pharmacother.

[32] Kim HY, Lee SY, Lee HS (2021). Beneficial effects of intravenous magnesium administration during robotic radical prostatectomy: a randomized controlled trial [J]. Adv Ther.

